# Spectrum and Inoculum Size Effect of a Rapid Antigen Detection Test for Group A Streptococcus in Children with Pharyngitis

**DOI:** 10.1371/journal.pone.0039085

**Published:** 2012-06-29

**Authors:** Jérémie F. Cohen, Martin Chalumeau, Corinne Levy, Philippe Bidet, Franck Thollot, Alain Wollner, Edouard Bingen, Robert Cohen

**Affiliations:** 1 Unité de Recherche Epidémiologique en Santé Périnatale et Santé des Femmes et des Enfants (U953), Inserm, Paris, France; 2 Service de Pédiatrie Générale, Hôpital Necker-Enfants-Malades, AP-HP et Université Paris-Descartes, Paris, France; 3 Association Clinique et Thérapeutique du Val-de-Marne (ACTIV), Saint-Maur-des-Fossés, France; 4 Service de Microbiologie, Hôpital Robert Debré, AP-HP et Université Paris-Diderot, Paris, France; 5 Association Française de Pédiatrie Ambulatoire (AFPA), Essey-lès-Nancy, France; 6 Service de Microbiologie, Centre Hospitalier Intercommunal de Créteil, Créteil, France; Centers for Disease Control & Prevention, United States of America

## Abstract

**Background:**

The stability of the accuracy of a diagnostic test is critical to whether clinicians can rely on its result. We aimed to assess whether the performance of a rapid antigen detection test (RADT) for group A streptococcus (GAS) is affected by the clinical spectrum and/or bacterial inoculum size.

**Methods:**

Throat swabs were collected from 785 children with pharyngitis in an office-based, prospective, multicenter study (2009–2010). We analysed the effect of clinical spectrum (i.e., the McIsaac score and its components) and inoculum size (light or heavy GAS growth) on the accuracy (sensitivity, specificity, likelihood ratios and predictive values) of a RADT, with laboratory throat culture as the reference test. We also evaluated the accuracy of a McIsaac-score–based decision rule.

**Results:**

GAS prevalence was 36% (95CI: 33%–40%). The inoculum was heavy for 85% of cases (81%–89%). We found a significant spectrum effect on sensitivity, specificity, likelihood ratios and positive predictive value (p<0.05) but not negative predictive value, which was stable at about 92%. RADT sensitivity was greater for children with heavy than light inoculum (95% vs. 40%, p<0.001). After stratification by inoculum size, the spectrum effect on RADT sensitivity was significant only in patients with light inoculum, on univariate and multivariate analysis. The McIsaac-score–based decision rule had 99% (97%–100%) sensitivity and 52% (48%–57%) specificity.

**Conclusions:**

Variations in RADT sensitivity only occur in patients with light inocula. Because the spectrum effect does not affect the negative predictive value of the test, clinicians who want to rule out GAS can rely on negative RADT results regardless of clinical features if they accept that about 10% of children with negative RADT results will have a positive throat culture. However, such a policy is more acceptable in populations with very low incidence of complications of GAS infection.

## Introduction

Group A streptococcus (GAS) is found in 20% to 40% of cases of childhood pharyngitis; the remaining cases are considered viral [1]. Clinical examination cannot distinguish accurately between viral and GAS pharyngitis [2], and a diagnostic test based on a throat swab is recommended in most countries [3]. Throat culture on a blood agar plate in a microbiology laboratory for 48 hours is the reference test for diagnosis of GAS pharyngitis [4]–[7]. Rapid antigen detection tests (RADTs) have been proposed as an alternative to throat culture. Compared to laboratory culture, RADTs have high specificity (≈95%) and results are immediate. Their main drawbacks are low sensitivity (≈85%, range 65.6% to 98.9%) [8], [9] and variations in sensitivity by clinical spectrum of the disease (spectrum effect, or spectrum bias) [10]–[12]. The major issue for the spectrum effect is the generalisability of test performance. First, the overall population estimate might not be generalisable to patient subpopulations; second, the diagnostic accuracy that was observed in one study might not be applicable to other patients. Three studies have shown a significant spectrum effect on RADT sensitivity, but these studies had methodological limitations suggesting selection, indication, partial verification, and measurement biases [13]–[15]. No studies investigated the effect of clinical spectrum on sensitivity, specificity, likelihood ratios and predictive values of RADTs at the same time [13]–[15]. As well, RADT sensitivity is affected by inoculum size [16]–[18] – the amount of GAS colonies identified on throat culture and considered a proxy of the bacterial load on the swab. In vitro, the threshold of positivity of RADTs is between 10^5^ and 10^7^ colony-forming units per ml [19]. To date, several studies evaluated the effects of clinical spectrum and inoculum size on RADT sensitivity, but these effects were studied only separately, and no study has analysed the potential relation of clinical spectrum and inoculum size.

Because of the need to reduce antibiotics consumption, clinical decision rules were developed to help clinicians determine which patients should undergo testing and/or treatment with antibiotics. A decision rule based on the McIsaac score was recently proposed for adults and children [20]–[22], but this McIsaac-score–strategy was insufficiently validated in children [21]–[23]. In one validation study by McIsaac *et al.*, the reader cannot evaluate the risk of selection bias because the distribution of scores in children was not reported [21]. Another validation study did not include children with low-risk scores (score ≤1) [22]. A third study aimed to validate the McIsaac score in children, but the rule was modified for application in low-resource settings and no longer integrated the use of a throat culture or RADT [23].

We aimed to determine whether the diagnostic accuracy of a RADT for GAS pharyngitis is affected by the clinical spectrum effect and/or bacterial inoculum size and to validate a McIsaac-score–based decision rule.

## Methods

### Study Design

This is a secondary analysis of data from an office-based, multicenter, prospective study that took place in France between October 2009 and June 2011 (unpublished data). The aims of the princeps study were to evaluate the frequency of GAS carriage in healthy children and to compare the performance of a RADT between children with pharyngitis and healthy children, with throat culture in a microbiology laboratory as the reference test (intermediate results presented at the 28^th^ Annual Meeting of The European Society for Paediatric Infectious Diseases, Nice, France, 2010; abstract A-229-0021-00920). This ancillary analysis was restricted to patients with pharyngitis from the first year of inclusion. The STARD statement was followed for reporting the results of the study [24].

### Patients

Eligible patients were 3 to 15 years old who were evaluated by their paediatrician between October 2009 and May 2010, who had a diagnosis of pharyngitis and did not receive antibiotics for 7 days before inclusion. Pharyngitis was defined by inflammation of the pharynx and/or tonsils. In total, 17 French paediatricians who are part of a research and teaching network (ACTIV) participated in the study [25].

### Throat Swabs

Throat samples were obtained by use of a double–swab collection–transportation system (Venturi Transystem Amies agar, COPAN Diagnostics, Corona, CA, USA). The RADT (StreptAtest, Dectrapharm, France), a GAS-specific immuno-chromatographic strip assay, was performed immediately with swab #1 collected in the paediatrician's office. Swab #2 was held at ambient temperature and sent within 72 hr to the Robert Debré Hospital laboratory by an express messenger service. On receipt, the swab was rolled over one-quarter of a trypticase soy agar plate with 5% sheep blood, and the inoculum was further distributed on the plate by streaking with a sterile wire loop. The plates were incubated anaerobically at 37°C and read after 18 to 24 hr. Negative cultures were reexamined after an additional 24 hr of incubation (48 hr total). ß-haemolytic colonies were further investigated by latex agglutination (Prolex, Pro-Lab Diagnostics, Richmond Hill, ON, Canada). With GAS positivity, inoculum size was estimated as follows: 1+, <10 colonies; 2+, 11–50 colonies; and 3+, >50 colonies per plate. Data for patients with 1+ and 2+ cultures were combined to produce a dichotomized variable (light or heavy GAS growth). Microbiologists were blinded to individual clinical data and RADT results.

### Clinical Data

We collected clinical data needed to calculate the McIsaac score for each patient. The McIsaac score predicts GAS pharyngitis and ranges from 0 to 5 [20]–[22]. McIsaac criteria include age 3 to 15 years, history of fever, tonsillar swelling or exudate, tender anterior cervical adenopathy, and absence of cough. As for other studies [13], [26], the adenopathy criterion was modified to include any anterior cervical adenopathy ≥1 cm and/or presence of tenderness because tender lymph nodes can be difficult to assess in children. As for other authors [13]–[15], extreme McIsaac scores were combined (scores 1 and 2, and 4 and 5), and for analysis of clinical components of the score, age was re-coded into 2 categories of equal range (3–8 years; 9–14 years).

### Sample Size

In the original study, sample size was estimated so that the 95% confidence interval (95CI) for RADT sensitivity would be a +/−5% estimation. Assuming sensitivity to vary between 75% and 95% and a GAS prevalence of 35%, a sample of 714 children with pharyngitis was needed. No other sample size calculation was performed for this secondary analysis.

### Ethics

The study protocol was approved by the Institutional Review Board Comité de Protection des Personnes Ile-de-France XI (n°09016) and the French administrative authorities (CNIL, n°1354254; Afssaps, n°2009-A00086-51). Parent and patient approval for participation was obtained before inclusion. Data were double entered into 4D software version 6.4 (4D) and the database was fully anonymized.

### Statistical Analysis

Hospital laboratory throat culture was considered the reference test. First, we described patient demographic features and overall prevalence of groups A, C and G β-haemolytic streptococci. Patients with group C or G β-haemolytic streptococci were considered GAS-negative in the analysis. Then, GAS prevalence by McIsaac score and each McIsaac criterion were described. Second, we used chi-square tests to investigate the clinical spectrum effect on RADT diagnostic accuracy (sensitivity, specificity, likelihood ratios and predictive values) by McIsaac score and each McIsaac criterion. Third, chi-square tests were used to compare the distribution of GAS inoculum size by office-to-laboratory delay (≤48 vs. >48 hr) and to study the effect of inoculum size (light or heavy GAS growth) on RADT sensitivity. Fourth, the distribution of heavy inocula by McIsaac score was explored by a chi-square test for trend, and the association of clinical signs and inoculum size was evaluated by comparing the frequency of heavy inoculum according to each clinical criterion by chi-square tests. Fifth, a binomial model with an identity link was used to evaluate the combination of clinical spectrum effect and inoculum size on RADT sensitivity [27]. Differences in RADT sensitivity were estimated by restricting the analysis to patients with a positive throat culture and using the result of the RADT as the outcome in the model. RADT sensitivity was investigated by univariate modeling according to each McIsaac criterion and then, multivariate modeling by all McIsaac criteria and inoculum size. Potential interaction between inoculum size and each McIsaac criterion was assessed by use of a Wald test. For multivariate modeling, variables were added one by one following the crescent univariate statistical significance order until non-convergence of the model. Significant interactions were managed by introducing corresponding interaction terms in the multivariate model and, in case of non-convergence, by stratification on inoculum size. Sixth, the accuracy of the following McIsaac-score–based decision rule was studied in terms of sensitivity and specificity: (a) scores <2, no further testing or antibiotic; (b) scores 2–3, culture all, antibiotics only with positive culture; (c) scores ≥4, treat empirically with antibiotics [22]. Statistical analysis involved use of Stata/SE 12 (StataCorp, College Station, TX, USA).

## Results

### Population Characteristics, GAS Prevalence

In total, 807 children met the inclusion criteria; 1 patient with unreadable RADT results, 2 with lost throat swabs and 19 with missing data for McIsaac score calculation were secondarily excluded. Therefore, analysis involved 785 children (351 girls [44.7%]), with mean (SD) age 6.1 (2.5) years. Overall, GAS prevalence was 36.3% (95CI, 32.9%–39.8%). In all, 4 (<1%) Group C and 7 (<1%) Group G β-haemolytic streptococci were identified on BAP. The mean age of secondarily excluded children was 5.9 (3.0) years and GAS was found in throat cultures for 4/20 (20.0% [7.9%–39.2%]).

GAS prevalence increased with increasing McIsaac score (p<0.001) (Tables 1 and 2), from 24.3% to 41.2% for children with ≤2 and ≥4 McIsaac score, respectively. Some clinical criteria were also associated with GAS: age <9 years, absence of cough, and presence of fever (Table 2).

**Table 1:** Distribution of the McIsaac scores and corresponding frequencies of positive results of rapid antigen detection test (RADT), group A streptococcus (GAS) and heavy GAS inocula (N  = 785).

	n (%)			
McIsaacscore^a^	Total	RADT-positive	GAS-positive^b^	Heavy inoculum^c^
1	12 (1)	2/12 (17)	3/12 (25)	2/3 (67)
2	103 (13)	21/103 (20)	25/103 (24)	20/25 (80)
3	262 (33)	82/262 (31)	89/262 (34)	73/89 (82)
4	286 (36)	115/286 (40)	114/286 (40)	100/114 (88)
5	122 (16)	54/122 (44)	54/122 (44)	48/54 (89)

a All scores were ≥1 because all included patients were 3 to 15 years old.

b Laboratory throat culture positive for GAS.

c p  = 0.09, χ^2^ test for trend for the frequency of heavy inoculum by McIsaac score.

**Table 2:** Spectrum effect analysis: performance of rapid antigen detection test by clinical spectrum.

	n (%)	GAS (%)	*p* a	Sensitivity (95CI)	*p* a	Specificity (95CI)	*p* a		PLR (95CI)	*p^b^*	NLR (95CI)		*p^b^*		PPV (95CI)		*p* a	NPV (95CI)			*p* a	
McIsaac score																						
≤2	115 (14)	24.3	<0.001^c^	75.0 (55.1–89.3)	0.02^c^	97.7 (91.9–99.7)	0.04^c^		32.6 (8.2–130.5)	0.23	0.26 (0.13–0.49)		0.09		91.3 (72.0–98.9)		0.61^c^	92.4 (84.9–96.9)			0.83^c^	
3	262 (33)	34.0		84.3 (75.0–91.1)		96.0 (91.8–98.4)			20.8 (10.0–43.3)		0.16 (0.10–0.27)				91.5 (83.2–96.5)			92.2 (87.3–95.7)				
≥4	408 (51)	41.2		89.9 (84.3–94.0)		92.5 (88.4–95.5)			12.0 (7.7–18.7)		0.11 (0.07–0.17)				89.3 (83.7–93.6)			92.9 (88.9–95.8)				
Age (years)																						
3–8	663 (84)	38.5	<0.01	89.0 (84.5–92.6)	<0.001	94.1 (91.4–96.2)	0.45		15.1 (10.2–22.4)	0.62	0.12 (0.08–0.17)		<0.001		90.4 (86.1–93.8)		0.48	93.2 (90.3–95.4)			0.26	
9–14	122 (16)	24.6		66.7 (47.2–82.7)		96.7 (90.8–99.3)			20.4 (6.5–64.0)		0.34 (0.21–0.57)				87.0 (66.4–97.2)			89.9 (82.2–95.0)				
Lack of cough																						
Yes	428 (55)	44.4	<0.001	90.0 (84.8–93.9)	0.02	94.1 (90.3–96.7)	0.65	15.3 (9.2–25.5)		0.89	0.11 (0.07–0.16)	0.02		92.4 (87.6–95.8)		0.07			92.2 (88.1–95.2)	0.76		
No	357 (45)	26.6		80.0 (70.5–87.5)		95.0 (91.7–97.3)		16.1 (9.4–27.7)			0.21 (0.14–0.31)			85.4 (76.3–92.0)					92.9 (89.2–95.7)			
Anterior cervical adenopathy																						
No	407 (52)	34.6	0.32	83.0 (75.7–88.8)	0.07	97.7 (95.2–99.2)	0.001		36.8 (16.6–81.4)	0.003	0.17 (0.12–0.25)		0.12		95.1 (89.7–98.2)		0.01	91.5 (87.7–94.5)			0.33	
Yes	378 (48)	38.1		90.3 (84.2–94.6)		91.0 (86.6–94.4)			10.1 (6.7–15.2)		0.11 (0.06–0.18)				86.1 (79.5–91.2)			93.8 (89.9–96.6)				
Tonsillar swelling or exudates																						
No	157 (20)	42.0	0.10	83.3 (72.1–91.4)	0.36	95.6 (89.1–98.8)	0.80		19.0 (7.2–49.7)	0.71	0.17 (0.10–0.30)		0.38		93.2 (83.5–98.1)		0.47	88.8 (80.8–94.3)			0.11	
Yes	628 (80)	34.9		87.7 (82.6–91.7)		94.4 (91.7–96.4)			15.6 (10.5–23.3)		0.13 (0.09–0.19)				89.3 (84.4–93.1)			93.5 (90.6–95.6)				
Fever																						
No	246 (31)	31.3	0.049	88.3 (79.0–94.5)	0.62	93.5 (88.7–96.7)	0.43		13.6 (7.6–24.2)	0.47	0.13 (0.07–0.23)		0.66		86.1 (76.5–92.8)		0.15	94.6 (90.0–97.5)			0.22	
Yes	539 (69)	38.6		86.1 (80.6–90.5)		95.2 (92.3–97.2)			17.8 (11.0–28.8)		0.15 (0.10–0.21)				91.8 (87.0–95.2)			91.6 (88.1–94.3)				
Total	785	36.3		86.7 (82.2–90.4)		94.6 (92.2–96.4)			16.1 (11.1–23.2)		0.14 (0.10–0.19)				90.1 (86.0–93.4)			92.6 (89.9–94.7)				

Abbreviations: GAS, group A streptococcus; PLR, positive likelihood ratio; NLR, negative likelihood ratio; PPV, positive predictive value; NPV, negative predictive value.

a χ^2^ test; ^b^ Mantel-Haenszel's χ^2^ test; ^c^ χ^2^ test for trend.

### Spectrum Effect

Overall, the sensitivity and specificity of the RADT was 86.7% (82.2%–90.4%) and 94.6% (92.2%–96.4%), respectively; positive and negative likelihood ratios 16.1 (11.1–23.2) and 0.14 (0.10–0.19), respectively; and positive and negative predictive values 90.1% (86.0%–93.4%) and 92.6% (89.9%–94.7%), respectively.

We found significant variations in RADT sensitivity and specificity by McIsaac score (Table 2). Sensitivity increased from 75% (55.1%–89.3%) to 84.3% (75.0%–91.1%) and to 89.9% (84.3%–94.0%; p  = 0.02), and specificity decreased from 97.7% (91.9%–99.7%) to 96.0% (91.8%–98.4%) and to 92.5% (88.4%–95.5%; p  = 0.04) with McIsaac scores ≤2, 3 and ≥4, respectively (Table 2). RADT likelihood ratios and predictive values varied, but not significantly, by McIsaac score.

The RADT performance varied significantly by McIsaac criteria. For example, sensitivity was significantly lower in children ≥9 years old than in younger children (66.7% [47.2%–82.7%] vs. 89.0% [84.5%–92.6%], p<0.01). The negative predictive value varied, but not significantly, by McIsaac score or clinical criteria, ranging from 88.8% (80.8%–94.3%) for children without tonsillar swelling or exudate to 94.6% (90.0%–97.5%) for children without fever.

### Effect of Inoculum Size on RADT Sensitivity

Among 285 GAS-positive throat cultures, 243 (85% [80.6%–89.2%]) showed heavy inoculum. The distribution of inoculum size did not differ by office-to-laboratory delay (≤48 vs. >48 hr, p  = 0.97). RADT sensitivity varied widely and significantly between patients with light and heavy inoculum (40.5% [25.6%–56.7%] vs. 94.7% [91.0%–97.1%], p<0.001). Light inoculum was found in 65.8% (50.0%–81.6%) of false-negative RADT cases versus 6.9% (3.7%–10.1%) of true-positive cases (p<0.001).

Frequency of heavy inocula increased with increasing McIsaac score, although not significantly (p  = 0.09, table 1). Heavy inoculum was significantly more frequent in children <9 years old than in older children (87.1% [82.9%–91.2%] vs. 70.0% [52.6%–87.4%], p<0.05).

### Combined Effect of Clinical Spectrum and Inoculum Size

The results of univariate binomial modeling for RADT sensitivity were almost identical to those of stratified analysis (Tables 2 and 3): age ≥9 years and presence of cough were significantly associated with decreased RADT sensitivity (p<0.05). After stratification on inoculum size, wide and statistically significant variations in RADT sensitivity were found in patients with light inoculum (e.g., 37.4% variation in sensitivity by age, p<0.01), but not widely (<6%) nor significantly in patients with heavy inoculum. Interactions were found between inoculum size and age (p  = 0.03), anterior cervical adenopathy (p  = 0.01), and tonsillar swelling or exudate (p  = 0.07).

**Table 3:** Analysis of combined spectrum and inoculum size effect on sensitivity of rapid antigen detection test (RADT).

	Univariate analysis^a^							Multivariate analysis^a^			
Variable	All inocula	*p*	Light inoculum	*p*	Heavy inoculum	*p*	*p* b	Light inoculum^c^	*p*	Heavy inoculum^d^	*p*
	(n = 285)		(n = 42)		(n = 243)			(n = 42)		(n = 243)	
Age (years)											
3–8	*ref*	0.01	*ref*	<0.01	*ref*	0.49	0.03	*ref*	<0.001	*ref*	0.36
9–14	−22.4 (−39.7; −5.1)		−37.4 (−64.1; −10.7)		−4.6 (−17.4; 8.3)			−40.9 (−57.9; −24.0)		−5.7 (−17.9; 6.4)	
Lack of cough											
Yes	*ref*	0.03	*ref*	0.09	*ref*	0.14	0.19	*ref*	<0.001	*ref*	0.08
No	−10.0 (−19.1; −9.0)		−25.0 (−53.6; 3.6)		−5.2 (−12.1; 1.7)			−23.6 (−23.7; −23.5)		−6.0 (−12.5; 0.6)	
Anterior cervical adenopathy											
No	*ref*	0.07	*ref*	0.01	*ref*	0.88	0.01	*ref*	0.01	*ref*	0.37
Yes	7.3 (−0.6; 15.2)		36.1 (7.7; 64.5)		−0.4 (−6.1; 5.2)			40.5 (16.7; 64.3)		2.0 (−2.3; 6.2)	
Tonsillar swelling or exudate											
No	*ref*	0.40	*ref*	0.12	*ref*	0.21	0.07	n/a^e^		*ref*	0.29
Yes	4.3 (−5.6; 14.3)		−25.0 (−56.2; 6.2)		5.4 (−3.1; 13.9)					4.0 (−3.4; 11.4)	
Fever											
No	*ref*	0.61	*ref*	0.27	*ref*	0.77	0.26	n/a^e^		*ref*	0.41
Yes	−2.3 (−10.8; 6.3)		−19.1 (−53.0; 14.8)		1.0 (−5.6; 7.6)					2.3 (−3.3; 8.0)	

Data RADT sensitivity difference (95% confidence interval) unless indicated.

Abbreviations: ref, reference; n/a, not applicable.

a Binomial modelling.

b Interaction test between clinical variables and inoculum size (Wald test).

c Model adjusted on age, lack of cough, anterior cervical adenopathy.

d Model adjusted on age, lack of cough, anterior cervical adenopathy, tonsillar swelling or exudate and fever.

e Not included in the model because of non-convergence.

Because of these multiple interactions and because of no convergence in the multivariate model after introducing interaction terms, 2 multivariate models of RADT sensitivity were fit by stratifying on inoculum size (Table 3). Results of multivariate modeling were close to those of univariate modeling. Light inoculum was associated with wide and statistically significant adjusted variation in RADT sensitivity (e.g., from 23.6% to 40.9%), but heavy inoculum was not widely (≤6%) or significantly associated.

### Performance of the McIsaac-score–based Decision Rule

Among 785 children, 522 (66%) would have received antibiotics according to the decision rule. The rule had 98.9% (97.0%–99.8%) sensitivity and 52.0% (47.5%–56.5%) specificity.

## Discussion

Accuracy of a diagnostic test can vary across patient subgroups within a population, a phenomenon referred to as spectrum effect (or spectrum bias) [10]–[12]. In assessing the relation of clinical spectrum and inoculum size with RADT performance for GAS pharyngitis, we confirm that the spectrum effect has an impact on RADT sensitivity and report for the first time that it also affects the specificity, likelihood ratios and positive predictive value of the test. However, the spectrum effect had no impact on the negative predictive value of the RADT, which remained stable at about 92%, regardless of McIsaac score or its components. Clinicians do not want to expose their patients to GAS suppurative and nonsuppurative complications by withholding antibiotic treatment and therefore need a diagnostic test with high, stable negative predictive value. In this population with a GAS prevalence of 36%, a patient with a negative RADT result had a probability of GAS-positive throat culture close to 8% regardless of clinical features.

This study confirms the important effect of inoculum size on RADT sensitivity, already described in other studies, and reports for the first time an association of clinical spectrum and inoculum size. Our data suggest that having more streptococci in the throat (greater inoculum size) might be associated with more intense symptoms (higher McIsaac score) (p  = 0.09), although we might not have had enough statistical power to validate this hypothesis. However, because we were not able to differentiate GAS carriers from truly infected patients (i.e., by assessment of streptococcal antibody response) [16], [28], we cannot conclude on whether these patients with low McIsaac scores and light inocula were more likely to be GAS carriers rather than truly infected patients.

We chose to investigate the McIsaac score in 3 categories rather than compare the accuracy of the RADT above and below a defined breakpoint [15] because this corresponds to the original aims of the score – to stratify patients into 3 levels of risk and to suggest a subsequent course of action (low-risk: no test, no antibiotics; intermediate risk: culture or RADT, antibiotics only for positive results; high-risk: empirical antibiotic treatment without testing) [20]–[22]. According to our results, the McIsaac score alone does not allow for ruling out or affirming the diagnosis of GAS pharyngitis because the reported GAS prevalence ranged from 25% (too-high level to rule out) to 44% (too-low level to affirm) in children with a score of 1 and 5, respectively. Moreover, the McIsaac-score–based decision rule had only 52.0% (47.5%–56.5%) specificity, which does not seem consistent with the current need to reduce antibiotic consumption to contain antimicrobial resistance.

One of the strengths of this study is its prospective, multicenter design, which limits the selection bias potentially present in other studies [13], [14]. All included children underwent RADT and laboratory culture, which excluded the possible indication and verification biases of other studies [13], [14]. The results of our study are close to those reported in the literature. GAS prevalence was 36.3% (32.9%–39.8%), which is close to that from a recent meta-analysis (37%, 32%–43%) [1]. The overall RADT sensitivity was 86.7% (82.2%–90.4%), which is close to that from another recent meta-analysis (85%, 84%–87%) [9]. A limitation is that we used a modified adenopathy criterion for the analyses. However, univariate and multivariate modeling results and the performance of the McIsaac-score-based decision rule were stable in a sensitivity analysis involving the original McIsaac adenopathy criterion instead of our modified criterion.

Another limitation to our study is that some throat swabs were plated >48 hr after collection. This delay had no significant effect on the distribution of GAS inocula, but prolonged or inadequate shipping conditions could have resulted in the loss of viability of GAS if the original swab had only small numbers of bacteria. Therefore, some swabs with light inocula could have been falsely read as negative on throat culture. Because RADTs are more likely to give negative results with light inocula, these swabs might have led to a systematic decrease in number of RADT false-negative results and systematic increase in number of RADT true-negative results, with over-estimation of the negative predictive value of the RADT as a result. We can assume that this bias occurred at random because shipping and all bacteriologic investigations were blinded to clinical data and RADT results – non-differential measurement bias. Such a bias usually leads to loss of power, and our results regarding the stability of the negative predictive value of the RADT should be considered with caution until they are confirmed with a larger sample of patients.

The American Academy of Pediatrics advises that “*Children with manifestations highly suggestive of viral infection […] generally should not be tested for GAS infection*” [7]. Such guidelines cannot be formally validated because the criteria proposed to determine patients who should be tested are not integrated in an explicit decision rule and remain subject to personal interpretation. Because of GAS carriage and because GAS complications have become uncommon in North America and Western Europe [29], we advocate that the target sensitivity of diagnostic strategies for GAS pharyngitis should not be 100%. Moreover, discussions should not focus on sensitivity alone. We showed that the negative predictive value of a test can be stable across patient subgroups because variations in prevalence can be balanced by concomitant variations in sensitivity and specificity. Therefore, clinicians who want to rule out GAS can rely on negative RADT results regardless of clinical features if they accept that about 10% of children with negative RADT results will have a positive throat culture. However, these results should be considered with caution because they depend highly on the study population, the training of clinical and laboratory personnel and the microbiological devices and protocols used. Moreover, available data suggest that false-negative RADT results are as likely to occur in truly infected patients as in GAS carriers [16], [28]. Therefore, such a policy would be more acceptable in populations with very low incidence of suppurative and non-suppurative complications of GAS infection [30]. The McIsaac-score–based decision rule is insufficiently accurate for children, and efforts are needed to develop more specific selective testing strategies for GAS pharyngitis in children.

## References

[1] Shaikh N, Leonard E, Martin JM (2010). Prevalence of streptococcal pharyngitis and streptococcal carriage in children: a meta-analysis.. Pediatrics.

[2] Shaikh N, Swaminathan N, Hooper EG (2011). Accuracy and Precision of the Signs and Symptoms of Streptococcal Pharyngitis in Children: A Systematic Review.. J Pediatr.

[3] Matthys J, De Meyere M, van Driel ML, De Sutter A (2007). Differences among international pharyngitis guidelines: not just academic.. Ann Fam Med.

[4] Bisno AL, Gerber MA, Gwaltney JM, Kaplan EL, Schwartz RH (2002). Practice guidelines for the diagnosis and management of group A streptococcal pharyngitis. Infectious Diseases Society of America.. Clin Infect Dis.

[5] Gerber MA (2005). Diagnosis and treatment of pharyngitis in children.. Pediatr Clin North Am 52: 729–747, vi.

[6] Choby BA (2009). Diagnosis and treatment of streptococcal pharyngitis.. Am Fam Physician.

[7] American Academy of Pediatrics, Pickering L, Baker C, Long S, McMillan J (2009). Group A Streptococcal Infections..

[8] Gerber MA, Shulman ST (2004). Rapid diagnosis of pharyngitis caused by group A streptococci.. Clin Microbiol Rev.

[9] Ruiz-Aragon J, Rodriguez Lopez R, Molina Linde JM (2010). Evaluation of rapid methods for detecting Streptococcus pyogenes. systematic review and meta-analysis.. An Pediatr.

[10] Mulherin SA, Miller WC (2002). Spectrum bias or spectrum effect? Subgroup variation in diagnostic test evaluation.. Ann Intern Med.

[11] Goehring C, Perrier A, Morabia A (2004). Spectrum bias: a quantitative and graphical analysis of the variability of medical diagnostic test performance.. Stat Med.

[12] Elie C, Coste J (2008). A methodological framework to distinguish spectrum effects from spectrum biases and to assess diagnostic and screening test accuracy for patient populations: application to the Papanicolaou cervical cancer smear test.. BMC Med Res Methodol.

[13] Hall MC, Kieke B, Gonzales R, Belongia EA (2004). Spectrum bias of a rapid antigen detection test for group A beta-hemolytic streptococcal pharyngitis in a pediatric population.. Pediatrics.

[14] Edmonson MB, Farwell KR (2005). Relationship between the clinical likelihood of group a streptococcal pharyngitis and the sensitivity of a rapid antigen-detection test in a pediatric practice.. Pediatrics.

[15] Tanz RR, Gerber MA, Kabat W, Rippe J, Seshadri R (2009). Performance of a rapid antigen-detection test and throat culture in community pediatric offices: implications for management of pharyngitis.. Pediatrics.

[16] Gerber MA, Randolph MF, Chanatry J, Wright LL, DeMeo KK (1986). Antigen detection test for streptococcal pharyngitis: evaluation of sensitivity with respect to true infections.. J Pediatr.

[17] Kuhn S, Davies HD, Katzko G, Jadavji T, Church DL (1999). Evaluation of the Strep A OIA assay versus culture methods: ability to detect different quantities of group A Streptococcus.. Diagn Microbiol Infect Dis.

[18] Kurtz B, Kurtz M, Roe M, Todd J (2000). Importance of inoculum size and sampling effect in rapid antigen detection for diagnosis of Streptococcus pyogenes pharyngitis.. J Clin Microbiol.

[19] Charlier-Bret N, Boucher B, Poyart C, Quesne G, Bingen E (2004). Rapid antigen detection tests for diagnosis of group A streptococcal pharyngitis: comparative evaluation of sensitivity and practicability of 16 in vitro diagnostics medical devices performed in July 2002 by the French health products safety agency (Afssaps) as part of its market control mission.. Pathol Biol (Paris).

[20] McIsaac WJ, White D, Tannenbaum D, Low DE (1998). A clinical score to reduce unnecessary antibiotic use in patients with sore throat.. CMAJ.

[21] McIsaac WJ, Goel V, To T, Low DE (2000). The validity of a sore throat score in family practice.. CMAJ.

[22] McIsaac WJ, Kellner JD, Aufricht P, Vanjaka A, Low DE (2004). Empirical validation of guidelines for the management of pharyngitis in children and adults.. JAMA.

[23] Fischer Walker CL, Rimoin AW, Hamza HS, Steinhoff MC (2006). Comparison of clinical prediction rules for management of pharyngitis in settings with limited resources.. J Pediatr.

[24] Bossuyt PM, Reitsma JB, Bruns DE, Gatsonis CA, Glasziou PP (2003). The STARD statement for reporting studies of diagnostic accuracy: explanation and elaboration.. Ann Intern Med.

[25] Cohen R, Levy C, Ovetchkine P, Boucherat M, Weil-Olivier C (2004). Evaluation of streptococcal clinical scores, rapid antigen detection tests and cultures for childhood pharyngitis.. Eur J Pediatr.

[26] Dimatteo LA, Lowenstein SR, Brimhall B, Reiquam W, Gonzales R (2001). The relationship between the clinical features of pharyngitis and the sensitivity of a rapid antigen test: evidence of spectrum bias.. Ann Emerg Med.

[27] Wacholder S (1986). Binomial regression in GLIM: estimating risk ratios and risk differences.. Am J Epidemiol.

[28] Johnson DR, Kurlan R, Leckman J, Kaplan EL (2010). The human immune response to streptococcal extracellular antigens: clinical, diagnostic, and potential pathogenetic implications.. Clin Infect Dis.

[29] Carapetis JR, Steer AC, Mulholland EK, Weber M (2005). The global burden of group A streptococcal diseases.. Lancet Infect Dis.

[30] Pelucchi C, Grigoryan L, Galeone C, Esposito S, Huovinen P (2012). Guideline for the management of acute sore throat.. Clin Microbiol Infect.

